# Monitoring of spontaneous pneumothorax using electrical impedance tomography: A case report

**DOI:** 10.1016/j.heliyon.2024.e25405

**Published:** 2024-02-01

**Authors:** Zhijun Gao, Lin Yang, Zhanqi Zhao, Meng Dai, Xinsheng Cao, Xuan Song, Binghua Zhang, Ke Zhao

**Affiliations:** a Department of Aerospace Medicine, Air Force Medical University, Xi'an, China; b School of Biomedical Engineering, Guangzhou Medical University, Guangzhou, China; c Department of Biomedical Engineering, Air Force Medical University, Xi'an, China; d Department of Pulmonary and Critical Care Medicine, 986th Hospital of Air Force, Air Force Medical University, Xi'an, China; e Department of Critical Care Medicine,Peking Union Medical College Hospital, Beijing, China

**Keywords:** Electrical impedance tomography, Bedside monitoring, Pneumothorax

## Abstract

Pneumothorax is an emergency in thoracic surgeries and respiratory medicine. A technique is warranted for real-time monitoring of pneumothorax at the bedside so that rapid diagnosis and timely intervention can be achieved. We report herein a case in which electrical impedance tomography (EIT) was employed at the bedside to monitor lung ventilation of a patient with spontaneous pneumothorax during treatment. It was found that the affected side/healthy side ventilation ratio and global inhomogeneity were strongly correlated with the severity of pneumothorax. The use of EIT allowed intuitive observation of the effect of pneumothorax on ventilation, which helped the doctors make immediate diagnosis and intervention. After timely and successful treatment, the patient was discharged. This is the first case reporting a complete therapeutic course of spontaneous pneumothorax assessed with EIT. Our case demonstrated that EIT could monitor regional ventilation loss of the affected side of pneumothorax patients at the bedside, and dynamically assess the treatment effect of pneumothorax, which provides an important imaging basis for clinical pneumothorax treatment.

## 1 Introduction

Pneumothorax refers to the abnormal accumulation of air in the pleural cavity causing lung collapse, which is an emergency in thoracic surgeries and respiratory medicine [1]. The pleural pressure remains negative relative to atmospheric pressure throughout the entire respiratory cycle in a healthy person, but the introduction of air into this space in spontaneity or due to injuries or any other causes will result in the development of pneumothorax [2]. The onset of pneumothorax may be potentially fatal depending on its pathogenesis [3,4].

Clinically, early diagnosis and management are urgently needed owing to the sudden change in intrapleural pressure, which will lead to the obstruction of venous return and affect the stability of cardiopulmonary circulation [5]. Currently, the examination and diagnosis of pneumothorax mainly rely on X-ray and CT scans that usually require to be frequently carried out to obtain definite pneumothorax condition throughout the period of diagnosis and treatment. However, the exposure to ionizing radiation and the need for specific operating sites must be considered before implementation. Thus, a technique that is non-invasive as well as capable of bedside and real-time monitoring is warranted. Electrical impedance tomography (EIT) can provide real-time images of lung ventilation with display of regional ventilation changes [6]. With EIT, lung ventilation can be intuitively observed in real time and pneumothorax can be detected at the bedside. Also, it has the advantages of non-radiation, high resolution and portability, which are conducive to rapid diagnosis and real-time monitoring of pneumothorax at the bedside. A recent retrospective study proposed to identify and monitor pneumothorax development with asymmetric non-ventilated regions captured with EIT [7]. Up to now, there has been no study demonstrating a prospective EIT application regarding this aspect.

In this report we present a case in which the lung ventilation of a patient with spontaneous pneumothorax was continuously monitored at the bedside during treatment. When the pneumothorax condition suddenly deteriorated, timely diagnosis and rapid treatment were administrated to the patient with the help of EIT. Improved pulmonary ventilation was observed after the early intervention. Informed consent of the case report was received from the subject prior to the report submission.

## 2 Case presentation

### 2.1 Patient information

A 25-year-old adult male (height:172 cm; weight: 68 kg; age: 25 years) suffer from spontaneous pneumothorax.

### 2.2 Clinical findings

EIT may be used to evaluate treatment process of pneumothorax at the bedside and has the potential to be an important auxiliary tool in the diagnosis and treatment of pneumothorax.

### 2.3 Diagnostic assessments

The patients visited our respiratory department (986th Hospital of Air Force) on June 21st, 2023, at around 08:00 due to dyspnea, chest pain, and chest tightness following severe coughing, which aggravated 7 hours after physical activity. The patient had no fever, headache, expectoration, hemoptysis, palpitation, abdominal pain, vomiting and other uncomfortable symptoms. The patient denied a history of hypertension, diabetes, coronary heart disease, drug and food allergy. Physical examination: body temperature, 36.2 °C; pulse, 113bpm; breathing, 18 bpm; blood pressure, 123/90 mmHg and clear mind, no cyanosis in the lips, no congestion in the pharynx, no enlargement of both tonsils. The patient's heart rate was 113 beats per minute, and there was no pathological murmur in each valve auscultation area. The patient's abdomen was flat and soft, there was no tenderness, rebound pain and muscle tension, the liver and spleen were not touched, Murphy's sign was negative, there was no percussion pain in liver, spleen and kidney, and there was no edema in both lower limbs. Above all, the tactile speech fibrillation of the patient's right chest was absent, the percussion was over-voiced, the respiratory sound of the right lung was low, and no dry-wet rales were heard. During auscultation, the vocal tactile fremitus of the right chest was absent, the percussion note was hyperresonant and the breath sounds of the right lung were absent or reduced. Chest X-ray indicated right pneumothorax with over 95 % compression of the right lung. By combining with patient history, clinical manifestations and auxiliary examination for comprehensive judgment, the preliminary diagnosis of the presented case is spontaneous pneumothorax (right).

### 2.4 Timeline and therapeutic intervention

After respiratory routine nursing, the patient underwent thoracic puncture and gas suction with local anesthesia. The patient sat backwards in the armchair with the second intercostal space of the right midline of the clavicle as the puncture point, routine iodophor disinfection of the skin, wearing aseptic gloves and laying aseptic hole towels. With 2 % lidocaine 5mL through the skin layer by layer local anesthesia to the pleura, the left hand fixed the puncture point skin, the right hand holding the chest puncture needle through the skin and subcutaneous tissue and then vertically pierced into the pleural cavity, indwelling guide wire, after skin expansion, placing the guide wire, pulling out the guide wire, and receiving a sterile syringe to extract the pleural gas 1000m1. Iodophor disinfection puncture point, covered with sterile gauze and fixed with adhesive tape. The operation went smoothly and there was no discomfort after the operation. Approximately 1000mL of pleural gas was withdrawn from the 2nd intercostal space of the right clavicular midline. Following the procedure, the patient's symptoms of shortness of breath and chest pain improved. Arterial blood gases improved on the second day. The patient was supported by high-flow nasal cannula (flow rate: 45L/min; FiO_2_:65 %; temperature: 33 °C). Treatment of infection was administrated with 2 g of ceftizoxime sodium twice daily.

On June 27th, the patient complained of shortness of breath and chest tightness again. After obtaining informed consent, non-invasive bedside EIT examination was performed (VenTom-100, MidasMED Biomedical technology, Suzhou, China). Firstly, selected an EIT electrode chest strap of appropriate size based on the patient's chest circumference (with 16 equidistant rubber electrodes distributed), and used alcohol cotton pads to disinfect the EIT electrode chest strap. Secondly, wear the EIT electrode chest strap on the patient's 4th ∼5 intercostal level, and made a mark on the patient's front and back midline to ensure that the wearing position is the same every time. EIT imaging showed obvious ventilation loss on the right lung's anterior side (right/left ventilation ratio (R/L) was only 0.35) and high inhomogeneity of the ventilation distribution (global inhomogeneity index (GI) was up to 0.67), indicating unsatisfactory treatment effect. A subsequent X-ray was requested by the doctor after reviewing the EIT image. The results confirmed right pneumothorax with right lung compression of approximately 70 %.

On June 28th, at 11:00, closed thoracic drainage was performed with local anesthesia, in which the patient was connected to a three-chamber system. The patient took the sitting position, took the second intercostal space of the midline of the right clavicle as the puncture point, disinfected the skin with routine iodophor, wore aseptic gloves, spread aseptic hole towels, used 2 % lidocaine 5mL local anesthesia epidermis to the pleural wall, cut open the skin incision 2cm, bluntly separated the hemostatic clamp to the parietal pleura, punctured the parietal pleural indwelling catheter (thick tube, 6.7mm diameter) and connected with 3-cavity water-sealed drainage bottle, iodophor disinfection puncture point, and adhesive tape fixed after covering with sterile gauze. The operation went smoothly. Following the procedure, the patient's symptoms of chest tightness and shortness of breath was ameliorated, and a small amount of sputum was coughed up. At 22:00, after routine checkup, it was found that no gas had escaped from the drainage bottle. The patient's shortness of breath symptoms was relieved. Bedside EIT showed that the ventilation area in the right lung had improved compared with that on the previous day (GI = 0.62), but the tidal variation on the right side was still significantly worse than that on the left (R/L = 0.36). According to the production of the patient's sputum, antibiotic treatment with moxifloxacin was administered empirically. On June 29th, bedside EIT imaging results indicated better global ventilation homogeneity and ventilation in the right lung compared with those on the previous day (GI = 0.57; R/L = 0.54), suggesting a desired response to antibiotic treatment.

On June 30th, bedside EIT results showed no significant change of ventilation in right lung compared with that on previous day (R/L = 0.53), and compression of the right lung tissue was still present compared with the left. Subsequent chest CT results revealed a compression of 15 % of the right lung tissue, which was significantly reduced from the result on June 27th and consistent with the EIT findings.

On July 2nd, during morning checkup, the patient reported aneliorated symptoms of shortness of breath and chest pain. The follow-up bedside EIT showed further improvement in right lung ventilation (R/L = 0.57), consistent with the patient's account. The three-chamber closed drainage tube was occluded for one day. From July 3rd to 5th, daily EIT follow-up demonstrated a gradual reduction in the proportion of compressed lung tissue (R/L = 0.35, 0.45, 0.87, respectively). With review of the EIT results, the doctor decided to occlude the drainage tube again.

On July 6th, EIT results revealed good ventilation in the right lung (R/L = 0.86) as well as good ventilation homogeneity (GI = 0.48), consistent with the result on the 5th, which indicated less compression of the right lung tissue and stable condition. Subsequent chest X-rays confirmed the almost disappearance of pneumothorax, with favorable re-expansion of the right lung. The patient was planned for discharge after another day of observation.

On the evening of July 7th, the patient suddenly experienced significant chest pain and shortness of breath. The next morning, bedside EIT results showed obvious ventilation deficiency in the right lung (R/L = 0.19) and poor ventilation homogeneity (GI = 0.74). Subsequent X-ray examination confirmed severe compression of the right lung tissue, approximately 80 %, consistent with the EIT findings. Consequently, continuous negative pressure three-chamber drainage was administered.

On July 10th, the patient reported alleviated symptoms, and EIT follow-up exhibited improved right lung ventilation (R/L = 0.48) and ventilation homogeneity (GI = 0.61). Chest X-rays revealed approximately 30 % compression of the right lung, and the drainage tube was clamped for the follow-up observation.

**Fig. 1:**
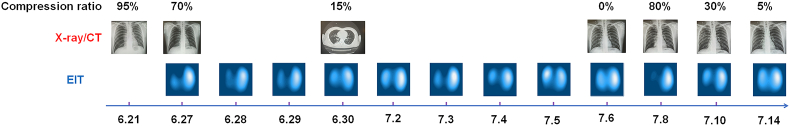
Timeline of patient treatment and examination. Tidal ventilation changes captured by electrical impedance tomography (EIT) and corresponding radiographic results in a patient with pneumothorax. Light blue regions in the EIT images indicate highly ventilated areas in the lung. (For interpretation of the references to colour in this figure legend, the reader is referred to the Web version of this article.)

**Fig. 2:**
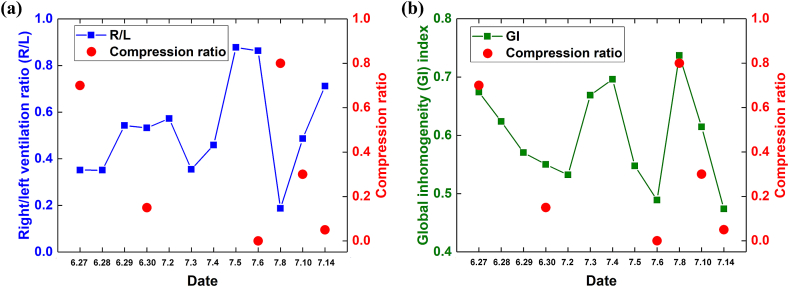
EIT-based indexes and compression ratio change over time in a patient with pneumothorax: (a) right/left ventilation ratio (R/L) and compression ratio; (b) global inhomogeneity (GI) index and compression ratio.

On July 14th, EIT follow-up showed near-normal ventilation in the right lung (R/L = 0.71) as well as ventilation homogeneity (GI = 0.47), and chest X-ray results suggested pneumothorax had mostly disappeared, with about 5 % compression of the right lung tissue. Given the improved condition of the patient, the closed chest drainage tube was removed. Since a small amount of gas could be absorbed on its own, the patient was allowed to be discharged. The schedule of clinical treatment and examination results of a patient with pneumothorax is shown in Table 1. The tidal ventilation changes captured by EIT throughout the patient's diagnosis and treatment are shown in Fig. 1, while the corresponding R/L and GI changes are shown in Fig. 2(a) and (b), respectively.

**Table 1:** Schedule of clinical treatment and examination results of a patient with pneumothorax.

	6.21	6.22	6.23	6.24–27	6.28	6.29	6.30	7.1–5	7.6	7.7–8	7.9–10	7.11–14
Clinical treatment	1000mL gas was extracted by thoracic puncture and aspiration, and anti-infective treatment (intravenous drip of cefazoxime 3g twice a day)	High-flow oxygen inhalation to promote gas excretion; continue anti-infective therapy (intravenous drip of cefazoxime 3g twice a day)	Continue high flow oxygen inhalation and exsufflation and anti-infective therapy (intravenous drip of cefazoxime 3g twice a day).	Continue high flow oxygen inhalation and anti-infection therapy (intravenous drip of cefotaxime 3g twice a day).	Closed thoracic drainage was performed with 3-cavity water-sealed drainage bottle and anti-infective treatment (intravenous drip of cefazoxime 3g twice a day).	Cefotaxime was discontinued and moxifloxacin (0.4 g intravenous drip 1/d) was given anti-infection therapy.	Continue anti-infective therapy (moxifloxacin 0.4g intravenous drip 1/day)	The drainage tube was clipped and observed for 1 day on the 2nd and 5th, and the anti-infective treatment was continued (moxifloxacin 0.4 g intravenous drip 1/day).	Anti-infective therapy meets the course of treatment and is discontinued.	Sudden chest tightness and shortness of breath on the evening of the 7th, open a 3-cavity water-sealed drainage bottle for negative pressure suction.	Continue closed drainage.	The drainage tube was clamped for observation on the 11th, pulled out on the 13th, and discharged on the 14th.
Examination results	Chest DR: right hydropneumothorax, right lung compression >95 %, blood gas analysis is generally normal: oxygen partial pressure 120 mmHg pressure, carbon dioxide partial pressure 35 mmHg, lactic acid 0.6mmol/L		Blood routine test: neutrophil ratio 75.2 %, neutrophil count 7.08–109 μ L, rest normal; myocardial enzyme, liver and kidney function, ion normal; hypersensitive C-reactive protein: 25.35mg/L.	27th re-examination of chest DR: right hydropneumothorax, right lung compression ≈95 %, right side less pleural effusion.			Chest CT plain scan: 1. Right hydropneumothorax (lung tissue is compressed by about 15 %); 2. Inflammation of the right lower lung.		Chest DR: no obvious abnormality was found in heart, lung and diaphragm.	8th chest DR: right hydropneumothorax, right lung compression ≈80 %, right a small amount of pleural effusion; 8th blood routine results are normal.	10th re-examination of the chest DR: right hydropneumothorax, right lung compression ≈30 %.	14th re-examination of the chest DR: the right pneumothorax, the right lung is compressed about 5 %.

### 2.5 Follow-up and outcomes

Weekly telephone follow-up for one month after discharge: the patient felt good and had no dyspnea and chest pain.

### 2.6 Patient perspective

The patient indicated that EIT provided a helpful means for himself to intuitively understand his pneumothorax condition.

## 3 Discussion

Pneumothorax is categorized as spontaneous pneumothorax and non-spontaneous such as traumatic with iatrogenic or no iatrogenic etiology [8]. Spontaneous pneumothorax is further divided into primary and secondary subtypes [9,10]. Primary spontaneous pneumothorax (PSP) occurs in patients with no obvious lung disease, while secondary spontaneous pneumothorax frequently occurs in elderly people with underlying lung conditions [11,12]. PSP commonly found in young, thin and tall males due to rupture of subpleural blebs or bullae [13]. The patient described in the present case was a young male (height:172 cm; weight: 68 kg; age: 25 years) without underlying pulmonary disease who complained of shortness of breath, chest pain, and chest tightness after a severe cough. Hence, the patient's pneumothorax was speculated to be PSP.

Spontaneous pneumothorax is regarded as a common and benign clinical entity. However, it can become a life-threatening condition especially if neglected and/or progresses to a tension pneumothorax because patients with a tension pneumothorax, which will result in not only respiratory but also cardiovascular compromise secondary to the decreased blood return to the heart from the decreased pressure gradient [14]. Consequently, immediate intervention is required to prevent the development of respiratory failure or obstructive shock from this “tension” physiology [15]. On the other hand, the choice of specific intervention (ranging from supplemental oxygen via nasal cannula to large-bore chest tube) is dependent upon the rapid assessment of pneumothorax condition [16]. Additionally, in the management of pneumothorax, continuous monitoring of pneumothorax condition is also needed to evaluate the treatment effect and trace the development of pneumothorax.

At present, chest X-ray examinations and CT scans were both alternative methods to confirm the diagnosis of pneumothorax [1,17,18]. Considering the radiation of X-ray and CT (0.013 mSv vs 3–10 mSv), EIT was attempted to continuously monitor lung ventilation at the bedside to reflect the severity of spontaneous pneumothorax in the present case. Interestingly, we found that bedside EIT could rapidly and intuitively exhibit the absence of regional ventilation caused by pneumothorax, and the index of right/left ventilation ratio (R/L) as well as GI was strongly correlated with the size of pneumothorax. These EIT results contributed significantly in the guidance for timely and effective treatment in this case. First, according to the EIT images, it was suggested that the initial treatment of gas extraction after hospital admission did not reduce the tissue compression degree of the pneumothorax as expected. As a result, the doctor decided to replace gas suction by closed thoracic drainage. Second, the continuous EIT results reflected the development of pneumothorax throughout the treatment period, which was beneficial for doctors to know the pneumothorax condition dynamically. Third, the sudden deterioration of pneumothorax was captured in time by EIT, resulting in further confirmation with X-ray, and immediate treatment was administrated for patient accordingly.

However, EIT has its known limitations in assessing pneumothorax: EIT image is unable to provide precise localization and size of pneumothorax owing to its characterization of two-dimensional tomographic imaging with a relatively low spatial resolution compared with that of CT or MRI [19,20]. In addition, many other possible factors may contribute to the non-ventilated regions in the lung, as opposed to pneumothorax. Nevertheless, our case demonstrated that, as a non-invasive and radiation-free tool that can provide real-time monitoring of lung ventilation at the bedside, EIT is able to dynamically monitor ventilation deficiency caused by pneumothorax and has the potential to be a complementary tool for clinical assessment of pneumothorax.

EIT was first proposed for pneumothorax monitoring in 2008. Researchers created pneumothorax models with experimental pigs by injecting air into the pleural cavity with a syringe. Characteristic changes were identified by EIT: increased electrical impedance (or brightness) at the end of exhalation in the pneumothorax area, with decreased ventilation ratio in the images of lung dynamic ventilation. EIT imaging is highly consistent with CT images in terms of pneumothorax location and size. The algorithm used can detect pneumothorax as small as 20 mL with a sensitivity of 100 % and specificity of 95 % [21]. Similar algorithms have been used to identify pneumothorax occurrences in other case reports [[22], [23], [24]]. In an animal experiment, EIT changes before and after pneumothorax in preterm lambs were observed, and in addition to increased end-expiratory impedance, “phase angle delay” was also observed, which might occur earlier than pneumothorax [25]. Other research groups used EIT to monitor pneumothorax development and recovery during lung re-expansion in ARDS patients successfully, suggesting EIT could be used to monitor pneumothorax occurrence, progression, and treatment effects [26]. However, one animal experiment indicated that the accuracy of pneumothorax diagnosis using EIT ventilation images was still low, relying solely on visual observation without baseline comparison or specific image post-processing [27]. In this case, the R/L ratio and GI index were used to evaluate the ventilation loss caused by pneumothorax throughout the entire treatment process. Future algorithm upgrades are expected to improve the accuracy and specificity of EIT in the diagnosis of pneumothorax at the bedside.

## 4 Conclusion

In conclusion, EIT can detect ventilation deficiency in the affected lung tissue of pneumothorax patients. Moreover, EIT has advantages such as non-invasiveness, radiation-free imaging and real-time monitoring. It may be used to evaluate treatment process of pneumothorax at the bedside and has the potential to be an important auxiliary tool in the diagnosis and treatment of pneumothorax.

## 5 Ethics statement

The study was conducted ethically in accordance with the World Medical Association Declaration of Helsinki. The patient provided written informed consent for publication of this case report and all the accompanying images.

## Funding statement

This study was supported by 10.13039/501100007128Natural Science Foundation of Shaanxi Province, China (2023-YBSF-130).

## Data availability statement

The data that support the findings of this study are available from the corresponding author upon reasonable request.

## Additional information

No additional information is available for this paper.

## CRediT authorship contribution statement

**Zhijun Gao:** Writing – review & editing, Data curation. **Lin Yang:** Writing – original draft, Conceptualization. **Zhanqi Zhao:** Writing – original draft, Formal analysis, Conceptualization. **Meng Dai:** Writing – review & editing, Software. **Xinsheng Cao:** Writing – review & editing, Project administration, Investigation. **Xuan Song:** Writing – review & editing, Methodology, Investigation. **Binghua Zhang:** Writing – review & editing, Validation, Supervision. **Ke Zhao:** Writing – review & editing, Data curation.

## Declaration of competing interest

The authors declare the following financial interests/personal relationships which may be considered as potential competing interests:Zhanqi Zhao reports a relationship with Drägerwerk AG & Co. KGaA that includes: consulting or advisory. Besides, he is an associate editor of the journal Heliyon but not involved in the decision of this manuscript.
